# XZH-5 Inhibits STAT3 Phosphorylation and Enhances the Cytotoxicity of Chemotherapeutic Drugs in Human Breast and Pancreatic Cancer Cells

**DOI:** 10.1371/journal.pone.0046624

**Published:** 2012-10-03

**Authors:** Aiguo Liu, Yan Liu, Zhigang Jin, Qun Hu, Li Lin, David Jou, Jing Yang, Zhenghu Xu, Hong Wang, Chenglong Li, Jiayuh Lin

**Affiliations:** 1 Department of Pediatrics, Tongji Hospital, Huazhong University of Science and Technology, Wuhan, Hubei, People's Republic of China; 2 Center for Childhood Cancer, The Research Institute at Nationwide Children's Hospital, Columbus, Ohio, United States of America; 3 Molecular, Cellular and Developmental Biology Program, The Ohio State University, Columbus, Ohio, United States of America; 4 Department of Comparative Biosciences, College of Veterinary Medicine, University of Illinois at Urbana-Champaign, Urbana, Illinois, United States of America; 5 Division of Cardiology, Department of Internal Medicine, Tongji Hospital, Huazhong University of Science and Technology, Wuhan, Hubei, People's Republic of China; 6 Department of Chemistry and Biochemistry, Miami University, Oxford, Ohio, United States of America; 7 Division of Medicinal Chemistry and Pharmacognosy, College of Pharmacy, The Ohio State University, Columbus, Ohio, United States of America; Sun Yat-sen University Medical School, China

## Abstract

Constitutive activation of Signal Transducers and Activators of Transcription 3 (STAT3) signaling is frequently detected in breast and pancreatic cancer. Inhibiting constitutive STAT3 signaling represents a promising molecular target for therapeutic approach. Using structure-based design, we developed a non-peptide cell-permeable, small molecule, termed as XZH-5, which targeted STAT3 phosphorylation. XZH-5 was found to inhibit STAT3 phosphorylation (Tyr705) and induce apoptosis in human breast and pancreatic cancer cell lines expressing elevated levels of phosphorylated STAT3. XZH-5 could also inhibit interleukin-6-induced STAT3 phosphorylation in cancer cell lines expressing low phosphorylated STAT3. Inhibition of STAT3 signaling by XZH-5 was confirmed by the down-regulation of downstream targets of STAT3, such as Cyclin D1, Bcl-2, and Survivin at mRNA level. In addition, XZH-5 inhibited colony formation, cell migration, and enhanced the cytotoxicity of chemotherapeutic drugs when combined with Doxorubicin or Gemcitabine. Our results indicate that XZH-5 may be a potential therapeutic agent for breast and pancreatic cancers with constitutive STAT3 signaling.

## Introduction

Cancer is a major public health problem in the United States, and one in four deaths is due to cancer. Breast cancer is the leading type of cancer affecting women. It is estimated that breast cancer accounts for over a quarter of all newly diagnosed cancer cases in women [1], [2]._ENREF_2 Pancreatic cancer is the fourth leading cause of cancer deaths in the United States. Diagnosis is followed by a poor prognosis, with a five year survival rate less than 5% [1], [3]._ENREF_2 The large number of cases and poor survival rate under current therapies necessitate the search for novel targeted therapies for cancer.

The Signal Transducers and Activators of Transcription (STAT) proteins are transcription factors that participate in relaying signals from growth factors and cytokines [4], [5]. STAT3 in particular, is constitutively active in a wide variety of human cancer cell lines and tissues, including breast and pancreatic cancer [2], [6], [7], [8], [9]. Constitutive STAT3 activity is required for the growth of cancer cells [10]. Due to its ability to promote malignancy, STAT3 is considered to be an oncogene [8], [11], [12]. Moreover, STAT3 deficient fibroblasts were resistant to transformation [13], [14], [15].

Using dominant-negative STAT3, STAT3 antisense oligonucleotides or RNA interference, blocking STAT3 signaling has provided further evidence of the potential of STAT3 as a target for cancer treatment [16], [17], [18], [19]. Inhibiting STAT3 has been successful, resulting in inducing growth arrest and apoptosis in different types of tumors. It was also determined that blocking STAT3 is neither harmful nor toxic to normal cells [14], [16]. Given the oncogenic functions, targeting STAT3 signaling represents a molecular therapeutic approach for cancer treatment.

**Figure 1:**
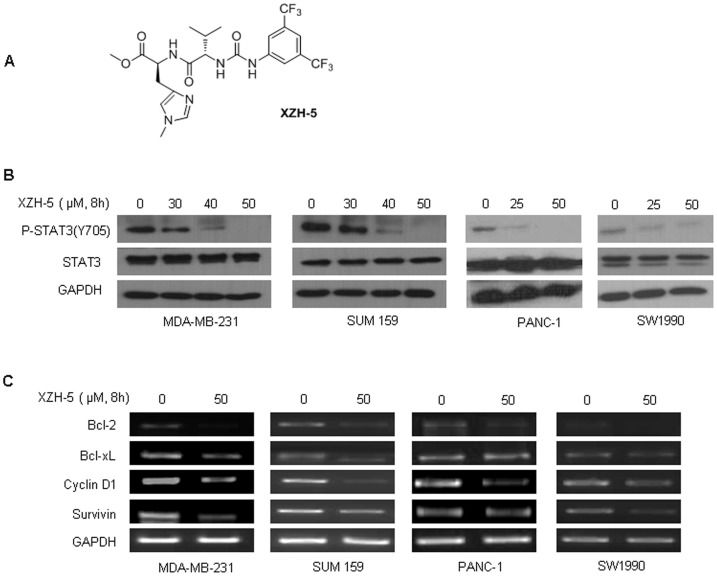
XZH-5 reduces STAT3 phosphorylation. (A) Structure of XZH-5. (B) MDA-MB-231, SUM 159, PANC-1, and SW1990 cells were treated with different concentrations of XZH-5 for 8 hours. p-STAT3 and STAT3 were analyzed by western blot. (C) MDA-MB-231, SUM 159, PANC-1 and SW1990 were treated with XZH-5 for 8 hours. The mRNA expression of BCL-2, BCL-xL, CyclinD1, Survivin, and GAPDH was analyzed by RT-PCR.

We used structure-based drug design to develop a STAT3 inhibitor, named XZH-5 (Figure 1A). Computer models with docking simulation showed that XZH-5 bound directly to the phosphorylated tyrosine 705 (pY705) binding site of the STAT3 monomer [9], [20]. We have shown previously that XZH-5 inhibits STAT3 phosphorylation and reduces cell viability in human HCC and rhabdomyosarcoma cells. In this study, we explored whether XZH-5 can inhibit STAT3 phosphorylation and induce apoptosis in human breast and pancreatic cancer cells that also express elevated levels of STAT3 phosphorylation. We demonstrated that XZH-5 inhibited STAT3 phosphorylation (Tyr705) and STAT3 activities, down-regulated STAT3 downstream targets, inhibited colony formation, cell migration, and induced apoptosis in human breast and pancreatic cancer cells. We also demonstrated that XZH-5 enhanced the cytotoxicity of Doxorubicin or Gemcitabine in breast and pancreatic cancer cells, respectively.

## Materials and Methods

### Cell Culture

Human breast cancer cell lines (MDA-MB-231, SUM159 and MCF-7), human pancreatic cancer cell lines (PANC-1, SW1990 and ASPC-1) and Hela cell line were purchased from the American Type Culture Collection (ATCC, Manassas, VA, USA). SUM159 cells were grown in Ham's F-12 medium (MediaTech Inc, Manassas, VA), Immortalized human pancreatic duct epithelial cells (HPDE) were provided by Dr Ming-Sound Tsao at the University of Toronto and were maintained in CnT-07CF epidermal keratinocyte medium (CELLnTEC Advanced Cell Systems, Bern, Switzerland) supplemented with 100 U/ml penicillin and 100 mg/ml streptomycin (Invitrogen Life Technologies, Carlsbad, CA) and 0.07 mM CaCl2 in addition to the manufacturer's provided supplements. The other cell lines were maintained in Dulbecco's Modified Eagle Medium (DMEM, MediaTech Inc). All the cell lines were cultured in medium supplemented with 10% fetal bovine serum (FBS, Invitrogen, Grand Island, NY, USA) and 100 U/ml penicillin/streptomycin B (MediaTech Inc), in a humidified 37°C incubator with 5% CO_2_.

### Western Blot Analysis

Cells were lysed in cold RIPA lysis buffer containing cocktail protease inhibitors to prepare whole-cell extracts. Lysates were then centrifuged at 14,000 rpm for 10 minutes to remove insoluble materials. 30–100 µg Protein samples were separated by SDS-PAGE and transferred onto PVDF member. After being blocked with 5% nonfat milk, the proteins were immunoblotted overnight at 4°C with 1∶1000 dilution of primary antibodies (Cell Signaling Technology, Beverly, MA, USA) against phospho-STAT3 (Tyrosine 705), STAT3, phospho-STAT1 (Tyrosine 701), STAT1, phospho-mTOR (Ser2448), phospho-JAK2 (Tyr1007/1008), P-AKT (Ser473), phospho-ERK1/2 (Thr202/Tyr204), cleaved caspase-3, and GAPDH, respectively, and 1∶10,000 dilution of HRP conjugated secondary antibody for 1 hour at room temperature. The target proteins were visualized by chemiluminescence (Cell Signaling Technology).

### Reverse Transcriptase-Polymerase Chain Reaction (RT-PCR)

MDA-MB-231, SUM159, PANC-1 and SW1990 cancer cells were treated with XZH-5 or DMSO at 60–80% confluence in the presence of 10% FBS for 8 hours. RNA from the cells was collected using RNeasy Kits (Qiagen, Valencia, CA, USA). PCR amplification was done under the following conditions: 5 min at 94°C followed by 25 cycles of 30 seconds at 94°C, 30 sec at 55°C, and 30 seconds at 72°C with a final extension of 5 min at 72°C. The DNA sequences of primers of STAT3 downstream target genes (CyclinD1, Survivin, Bcl-xL and Bcl-2) used for RT-PCR analysis were shown in Table S1.

### Apoptosis Assay

Apoptosis was measured using caspase3/7 assay (Promega, Madison, WI, USA) according to the manufacturer's manual. MDA-MB-231, SUM159, PANC-1 and SW1990 cells were seeded into a 96-well plate and treated as indicated in cell viability assay. After the treatment with different concentration of XZH-5 for 2 or 8 hours, 100 µl of Apo-One Caspase3/7 reagent was added to each well and the plate was incubated at 37°C for 30 min. The fluorescence was measured at an excitation wavelength range of 485 nm and an emission wavelength range of 530 nm.

### IL-6-induced STAT3 phosphorylation and IFN-γ-induced STAT1 phosphorylation

MCF-7 breast cancer cells and ASPC-1 pancreatic cancer cells which express low level of p-STAT3 were seeded in 10 cm plates and allowed to adhere overnight Then the cells were cultured in serum free medium for 24 hours and were pretreated with 25 µM or 50 µM of XZH-5 for 2 hours, followed by 50 ng/ml of IL-6 or IFN-γ for 30 minutes. The cells were harvested and analyzed by western blot for p-STAT3 or p-STAT1.

### Immunofluorescence

MCF-7 cells seeded on glass slides in 6-well plate were cultured in serum-free medium for 24 hours. Then, cells were pretreated with XZH-5 (50 µM) for 2 hours followed by IL-6 (50 ng/ml) for 30 min, and were fixed with cold methanol for 15 min. After washing in phosphate- buffered saline (PBS), the slides were blocked with 5% normal goat serum and 0.3% Triton X-100 in PBS (PBST) for 1 hour. The slides were incubated with primary antibodies against p-STAT3 or STAT3 proteins (Cell Signaling Technology) overnight at 4°C. Then, the slides were washed with PBST and incubated with goat anti-rabbit FIFC-conjugated secondary antibody (Invitrogen). The cells were mounted with Vectashield Hardset mounting medium with DAPI (Vector Laboratories, Burlingame, CA, USA). Photomicrographs were captured by Leica Microsystems (Bannockburn, IL, USA).

### Wound Healing/Cell Migration Assay

MDA-MB-231 and PANC-1 cells were seeded in a six-well plate. When the cells were 100% confluent, the monolayer was scratched using a pipette tip and washed once to remove non-adherent cells. New medium in the presence of 10% FBS containing XZH-5 (25 µM or 50 µM) was added. The treatment was removed after 2 hours and fresh medium was added. After 48 hours without treatment, the cells were observed under the microscope. Pictures were captured by Leica Microsystems.

### Colony Formation Assay

MDA-MB-231 and PANC-1 cells were seeded in DMEM with 10% FBS for 24 hours, then pre-treated with 25 µM or 50 µM of XZH-5 at 60–80% confluent for 2 hours. After being digested, 1000 cells per 100 mm dish were seeded in fresh medium to grow for 14 days to form colonies, which were then stained with crystal violet (Fisher Chemical, Fair Lawn, NJ, USA). The colony numbers were counted to determine long term effects of XZH-5.

### Cell Viability Assay

Cell viability was measured using CyQUANT NF Kit (Invitrogen). Cells were seeded into a 96-well plate. MDA-MB-231 cells were treated with Doxorubicin with different concentrations of XZH-5. PANC-1 cells were treated with Gemcitabine with different concentrations of XZH-5. After the treatment, medium was removed, and 100 µl of dye solution was added to each well. The plate was incubated at 37°C for 30 minutes, and the fluorescence was measured at an excitation/emission wavelength range of 485/530 nm.

### Cell Transfection and Luciferase Assay

Hela cells were cultured in DMEM containing 10% fetal bovine serum, penicillin and streptomycin, at 37°C and 5% CO_2_. Cell transfection was performed with Lipofectamine 2000. For luciferase assay, a Stat3 binding site-dependent luciferase reporter (Stat3-Luc) was used to monitor Stat3 transcriptional activity. As control, AP1-Luc was used to monitor AP1 transcriptional activity and pGL3-Promoter (Promega) which contains cDNA encoding luciferase under control of SV40 promoter was used to monitor SV40 promoter activity. Cells were transfected with 400 ng luciferase constructs and 50 ng pRL-tk which was used to normalize transfection efficiency. One day after cell transfection, cells were treated with DMSO or XZH-5 with indicated concentration for 8 hours. Luciferase activities were measured in duplicate or triplicate with passive lysis buffer by using the dual-luciferase reporter assay system (Promega).

### Animal Experiment

All animal studies were conducted in accordance with the principles and standard procedures approved by IACUC at the Research Institute at Nationwide Children's Hospital. 4- to 5-week-old female non-obese diabetic/severe combined immunodeficiency (NOD/SCID) mice were purchased from Jackson Laboratory (Bar Harbor, ME). DMSO or 100 mg/kg of XZH-5 was injected subcutaneously every other day for two weeks. Body weight and death incidence were monitored.

## Results

### XZH-5 inhibits STAT3 phosphorylation and induces apoptosis

To examine whether XZH-5 may inhibit STAT3 phosphorylation in breast cancer and pancreatic cancer cells expressing persistently activated STAT3, MDA-MB-231, SUM159, PANC-1, and SW1990 cells were treated with two different concentrations of XZH-5 as indicated for 8 hours. Phosphorylated STAT3 was reduced in a XZH-5 dose-dependent fashion (Figure 1B), and no effects were observed on total STAT3.

XZH-5 treatment decreased STAT3 transcriptional activity but had little or no effect on AP1 and pGL3, respectively (Figure S1). To further analyze whether the treatment of XZH-5 may affect the expression of STAT3 downstream genes, we looked at the mRNA levels of bcl-2, Bcl-xL, cyclinD1, and Survivin in XZH-5 treated cells. In MDA-MD-231 and SUM159 cells, all four STAT3 downstream targeted genes were downregulated. In PANC-1 cells, Bcl-2 and Cyclin D1 were decreased, and in SW1990 cells, only Survivin was decreased by XZH-5 (Figure 1C).

**Figure 2:**
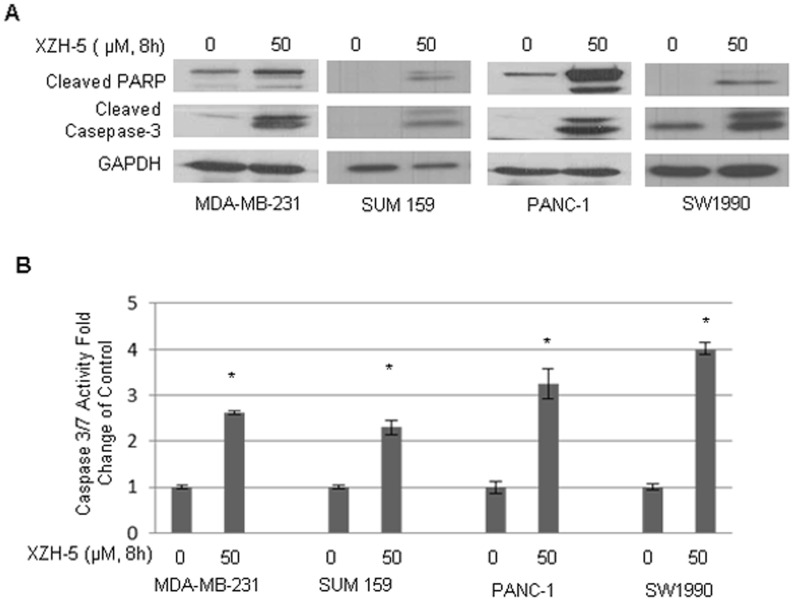
XZH-5 induces apoptosis. (A) MDA-MB-231, SUM 159, PANC-1 and SW1990 cells were treated with XZH-5 for 8 hours. Cleaved PARP and Cleaved Caspase-3 were analyzed by western blot. (B) Caspase-3/7 activity was measured in XZH-5 treated MDA-MB-231, SUM 159, PANC-1 and SW1990 cells. The data represented three independent results.

We also observed that decreases STAT3 phosphorylation were concomitant with increased cleaved PARP and Caspase-3 levels (Figure 2A). In addition to the increased levels of cleaved Caspase-3, the activity of Caspase-3/7 was also enhanced in XZH-5 treated cells (Figure 2B).

To rule out the possibility that XZH-5 also affect other signaling pathways, we examined mTOR, JAK2, AKT, and ERK pathway. Our data showed that XZH-5 treatment did not affect these signaling pathways (Figure S3).

While XZH-5 treatment induced apoptosis in cancer cells, it did not affect the viability of normal cells at the same concentrations (Figure S2A). Although higher concentration of XZH-5 was used, it did not lead to body weight loss in mice (Figure S2B). Both results suggest low toxicity of XZH-5.

### XZH-5 inhibits IL-6 stimulated STAT3 phosphorylation and nuclear accumulation

**Figure 3:**
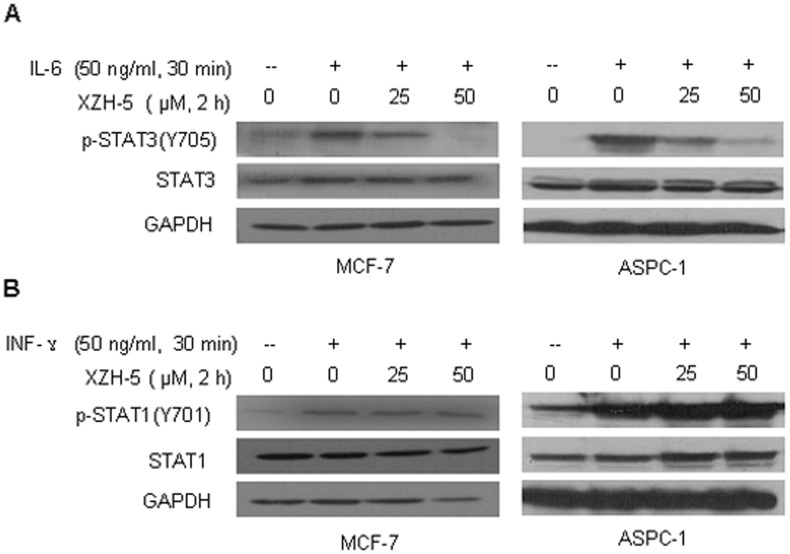
XZH-5 inhibits IL-6-induced STAT3 phosphorylation. MCF-7 and ASPC-1 cells were pre-treated with XZH-5 for 2 hours, followed by 50 ng/ml of IL-6 (A) or IFN-γ (B). p-STAT3 (A) and p-STAT1 (B) were analyzed by western blot.

IL-6 has been shown to induce STAT3 phosphorylation and may play a role in cancer development [21], [22], [23], [24], [25]. We examined whether XZH-5 may inhibit IL-6-induced STAT3 activation. MCF-7 and ASPC-1 cancer cells, which expressed low level of phosphorylated STAT3, were pre-treated with XZH-5 for 2 hours followed by the addition of IL-6. IL-6 stimulated STAT3 phosphorylation, whereas XZH-5 pre-treatment blocked IL-6-induced STAT3 activation (Figure 3A). Compared to the inhibitory effects on IL-6-induced STAT3 phosphorylation, XZH-5 did not affect IFN-γ-induced STAT1 phosphorylation (Figure 3B). These results suggest that XZH-5 selectively inhibits IL-6-induced STAT3 phosphorylation.

**Figure 4:**
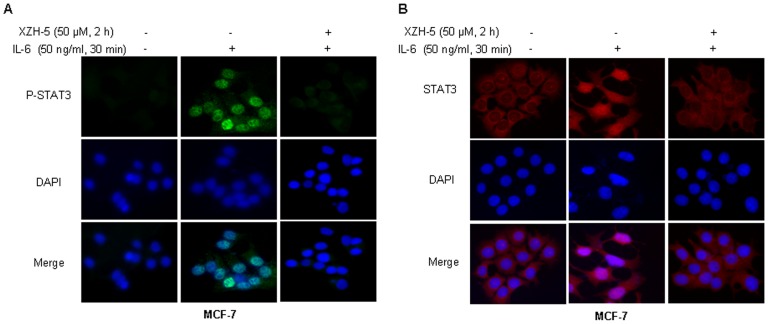
XZH-5 blocks the IL-6-induced p-STAT3 nuclear accumulation and STAT3 nuclear translocation. MCF-7 cells were pre-treated with 50 µM of XZH-5 for 2 hours followed by 50 ng/ml of IL-6 for 30 minutes. After the treatment, the localization of p-STAT3 (A) and STAT3 (B) was analyzed by immunofluorescence.

We then examined whether XZH-5 pre-treatment may block IL-6-induced P-STAT3 nuclear accumulation and STAT3 nuclear translocation. MCF-7 cells were pre-treated with 50 µM of XZH-5 for two hours followed by 50 ng/ml of IL-6 for 30 minutes. After the treatment, immunofluorescence was performed to analyze the localization of P-STAT3 and STAT3. IL-6 induced the accumulation of P-STAT3 in the nucleus, whereas the pre-treatment with XZH-5 blocked this process (Figure 4A). In addition, most STAT3 molecules were in the cytoplasm in the absence of IL-6. IL-6 treatment resulted in STAT3 nuclear translocation, which was blocked by XZH-5 pre-treatment (Figure 4B).

### XZH-5 reduces colony formation and migration

**Figure 5:**
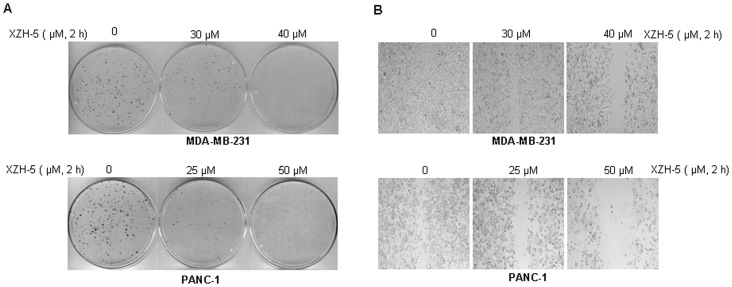
XZH-5 reduces colony forming ability and inhibits cell migration. (A) MDA-MB-231 and PANC-1 cells were treated with XZH-5 for 2 hours. After the treatment, viable cells were counted and the same number of cells were seeded and cultured for two week. Colonies were fixed by ice-cold methanol and were stained by 1% crystal violet. (B) When MDA-MB-231 and PANC-1 cells were 100% confluent, the monolayer was scratched using a pipette tip. Then the cells were treated with XZH-5 or DMSO for 2 hours. After the treatment, fresh medium was added and cells were cultured for 48 hours.

We next sought to investigate whether XZH-5 treatment may inhibit the colony formation capability. Since 2 hour-treatment inhibited STAT3 phosphorylation (Figure 3) but did not change the levels of apoptosis (Figure S4A and S4B) and cell viability (Figure S4C), we treated MDA-MB-231 and PANC-1 cells with XZH-5 for 2 hours. After the treatment, the same number of viable cancer cells was seeded and cultured in fresh medium without XZH-5 for two weeks. As shown in Figure 5A, XZH-5 treatment remarkably reduced colony formation capability.

STAT3 has been shown to be involved in wound-healing and migration of cancer cells which may lead to invasion and metastasis. We evaluated whether XZH-5 may affect cell migration. When cells were 100% confluent, the monolayer was scratched using a pipette tip. Then the cells were treated with XZH-5 or DMSO for two hours. After 48 hours, we observed that XZH-5 treatment reduced migration ability (Figure 5B).

#### XZH-5 enhances the cytotoxicity of chemotherapeutic drugs when combined with other anti-cancer drugs

**Figure 6:**
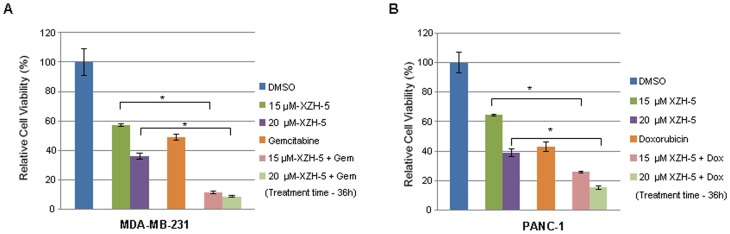
XZH-5 enhances cytotoxicity of chemotherapeutic drugs when combined with other anti-cancer drugs. (A) MDA-MB-231 cells were treated with 2.5 µM of Doxorubicin with or without 15 µM and 20 µM of XZH-5. (B) PANC-1 cells were treated with 250 nM of Gemcitabine with or without 15 µM and 20 µM of XZH-5. After 36 hours, cell viability was measured by fluorescence.

To investigate whether XZH-5 would lead to more cell death when combined with other anti-cancer drugs, cells were seeded in 96-well plates in triplicate at a density of 3,000 cells per well. We treated MDA-MB-231 cells with 2.5 µM of Doxorubicin (Figure 6A) and PANC-1 cells with 250 nM of Gemcitabine (Figure 6B) combined with 15 µM or 20 µM of XZH-5 for 36 hours, cell viability was measured. The combined treatment significantly reduced viable cell numbers in the presence of XZH-5, indicating synergism between XZH-5 and Doxorubicin or Gemcitabine.

## Discussion

Breast cancer is the most frequent carcinoma in females and the second most common cause of cancer-related mortality in women. Pancreatic cancer ranks fourth among cancer-related death in the United States. Breast cancer and pancreatic cancer are estimated to cause approximately 40,230 and 36,800 deaths respectively for the year ending in 2010 [1]. Selectively targeting activated STAT3 signaling has been shown to be effective in inhibiting cancer-associated processes and cancer cell viability [26], [27]. In our previous studies, we have reported the inhibitory effects of XZH-5 on rhabdomyosarcoma and liver cancer [9], [20]. It was unclear whether it could also inhibit STAT3 phosphorylation in breast and pancreatic cancer. Herein we investigated the effects of XZH-5 on breast and pancreatic cancer cell lines.

In this study, we demonstrated that XZH-5 could inhibit STAT3 phosphorylation in pancreatic and breast cancer cell lines expressing constitutive STAT3. The inhibition of STAT3 by XZH-5 was confirmed by the down regulated STAT3 downstream genes, such as Bcl-2, Bcl-xL, Cyclin D1, and Survivin. Meanwhile, the treatment with XZH-5 induced apoptosis and reduced the colony forming ability as well as cell migration.

In addition to inhibiting constitutively activated STAT3, the pre-treatment with XZH-5 could also block IL-6-induced STAT3 phosphorylation. IL-6 is a major mediator of inflammation and STAT3 activator and may prevent apoptosis to keep cells alive under very toxic conditions [28]. In this study, we showed that Exogenous IL-6 induced STAT3 phosphorylation and nuclear translocation. XZH-5 pre-treatment blocked this process, whereas it did not affect IFN-γ-induced STAT1 phosphorylation.

To address whether disruption of STAT3 will lead to chemosensitivity in cancer cells, we inhibited STATs activation with XZH-5 in the presence of other chemotherapeutic drugs. Doxorubicin, a topoisomerase II inhibitor and Gemcitabine, a nucleoside analog, are most active drugs used in various carcinomas including breast and pancreatic cancer. More and more relapse or resistance has been reported in clinic. On the other hand, patients could not endure chemotherapy due to drugs' side effect. Our study indicated that XZH-5 enhanced Doxorubicin and Gemcitabine cytotoxicity in breast and pancreatic cancer cells, respectively. This may be due to inhibiting the secretion of growth factors and cytokines, such as IL-6. In addition to growth factors and cytokines up-regulated by STAT3, Bcl-2, Bcl-xL, and Survivin also contribute to anti-apoptotic activity. Through blocking the expression of Bcl-2, Bcl-xL, and Survivin with XZH-5, cancer cells displayed enhanced chemosensitivity. Thus, we could decrease the dosages of these drugs and then reduce their toxicity when combined with XZH-5.

All through this study, we used different concentration of XZH-5. To investigate whether XZH-5 binds to STAT3 to inhibit its phosphorylation, we also use IL-6 to activate STAT3. Since IL-6 can turn on STAT3 pathway in 30 minutes, to avoid apoptosis, we pre-treated cells with XZH-5 for 2 hours followed by IL-6 treatment. To determine whether XZH-5 can inhibit STAT3 and cause apoptosis, we treated cells with XZH-5 for 8 hours. To examine whether combing XZH-5 with other drugs will result in more cell death, we treated cells for 36 hours to allow other drugs to take effect.

In conclusion, we have demonstrated that XZH-5 is an effective STAT3 inhibitor. These results provide the supportive evidence that XZH-5 may be effective to suppress pancreatic and breast tumor cell growth in cancer patients with constitutive STAT3 signaling.

## Supporting Information

Figure S1 **XZH-5 decreases STAT3 transcriptional activity.** XZH-5 treatment decreased STAT3 transcriptional activity but had little or no effect on AP1 and pGL3, respectively.
(TIF)

Figure S2 **XZH-5 shows low toxicity.** XZH-5 did not affect the viability of normal cells at the same concentrations (A). High concentration of XZH-5 did not lead to body weight loss in mice (B).
(TIF)

Figure S3 **XZH-5 does not affect other signaling pathways.** mTOR, JAK2, AKT, and ERK pathways were examined, Our data showed that XZH-5 treatment did not affect these signaling pathways .
(TIF)

Figure S4 **Two hour-treatment does not induce apoptosis and does not decrease cell viability significantly.** MDA-MB-231 and PANC-1 cells were treated by XZH-5 for 2 hours. After treatment, cleaved caspase 3 (A) and caspase 3/7 activity (B) were analyzed. Cell viability was also measured (C).
(TIF)

Table S1 **The DNA sequences of primers of STAT3 downstream target genes.**
(TIF)
