# Supplementary figures and images for: XZH-5 Inhibits STAT3 Phosphorylation and Enhances the Cytotoxicity of Chemotherapeutic Drugs in Human Breast and Pancreatic Cancer Cells
Source: PLoS One. 2012 Oct 3;7(10):e46624. doi: 10.1371/journal.pone.0046624 (PMC3463519; doi:10.1371/journal.pone.0046624)

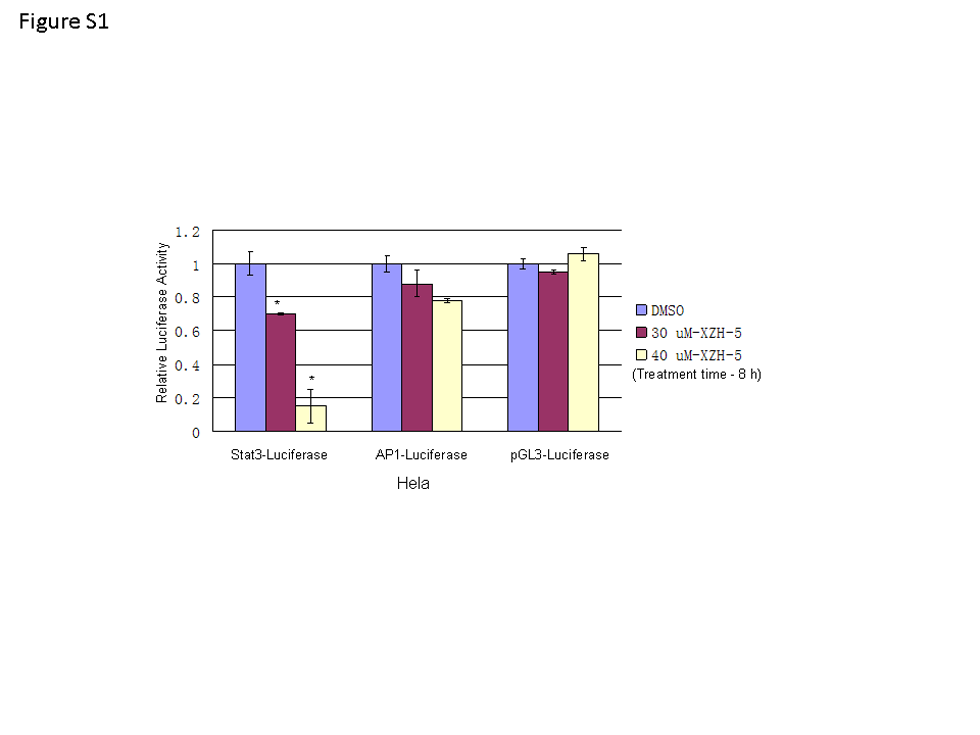

Supplement: Figure S1 — XZH-5 decreases STAT3 transcriptional activity. XZH-5 treatment decreased STAT3 transcriptional activity but had little or no effect on AP1 and pGL3, respectively. (TIF) [file pone.0046624.s001.tif]

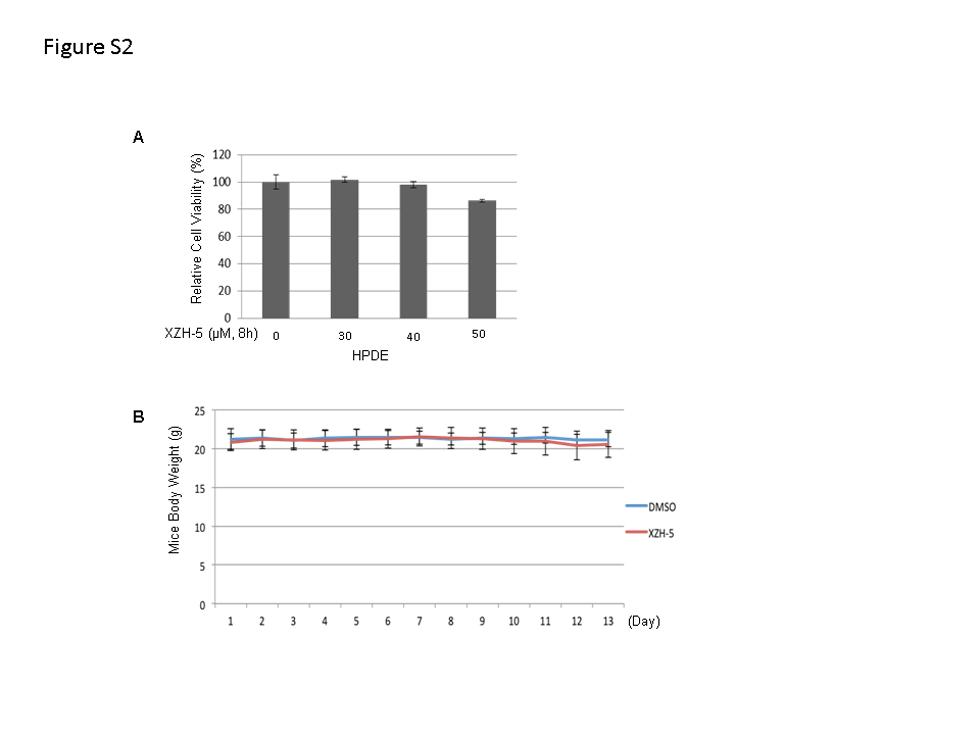

Supplement: Figure S2 — XZH-5 shows low toxicity. XZH-5 did not affect the viability of normal cells at the same concentrations (A). High concentration of XZH-5 did not lead to body weight loss in mice (B). (TIF) [file pone.0046624.s002.tif]

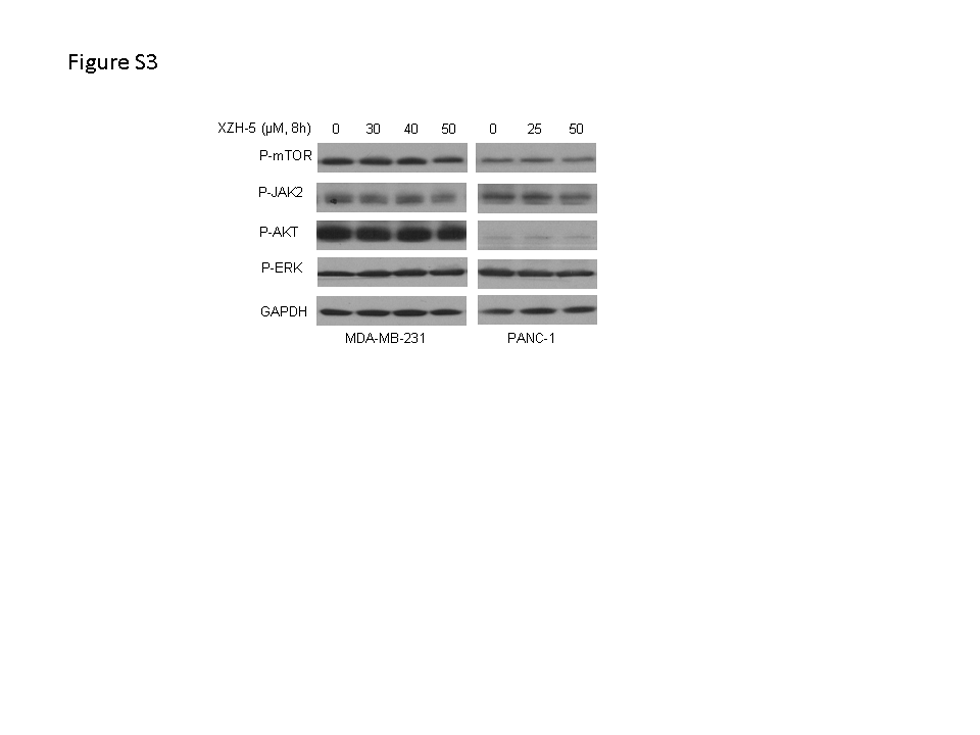

Supplement: Figure S3 — XZH-5 does not affect other signaling pathways. mTOR, JAK2, AKT, and ERK pathways were examined, Our data showed that XZH-5 treatment did not affect these signaling pathways . (TIF) [file pone.0046624.s003.tif]

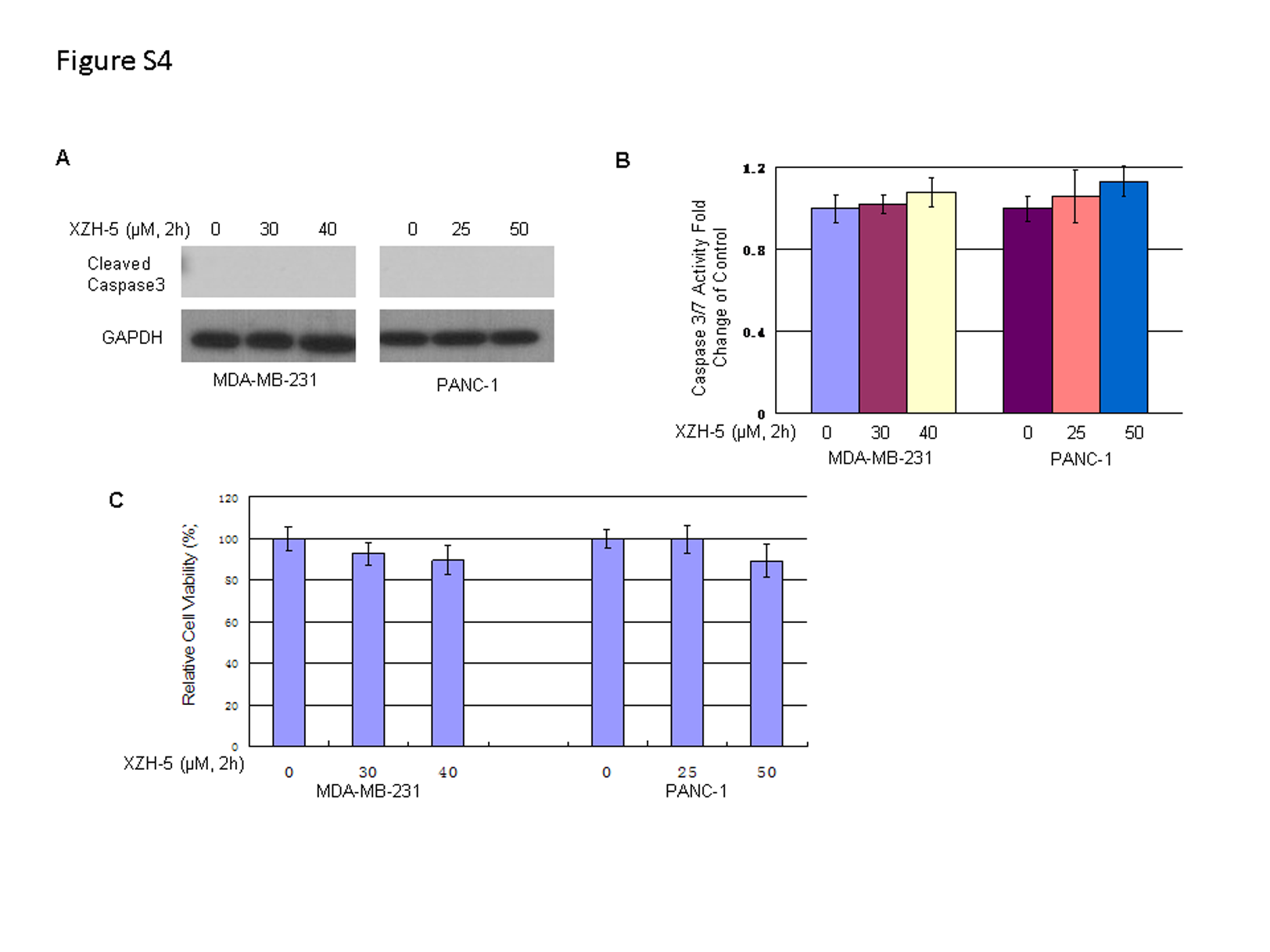

Supplement: Figure S4 — Two hour-treatment does not induce apoptosis and does not decrease cell viability significantly. MDA-MB-231 and PANC-1 cells were treated by XZH-5 for 2 hours. After treatment, cleaved caspase 3 (A) and caspase 3/7 activity (B) were analyzed. Cell viability was also measured (C). (TIF) [file pone.0046624.s004.tif]
